# Frequency-dependent ERK phosphorylation in spinal neurons by electric stimulation of the sciatic nerve and the role in electrophysiological activity

**DOI:** 10.1186/1744-8069-3-18

**Published:** 2007-07-16

**Authors:** Tomokazu Fukui, Yi Dai, Koichi Iwata, Hiroshi Kamo, Hiroki Yamanaka, Koichi Obata, Kimiko Kobayashi, Shenglan Wang, Xiuyu Cui, Shinichi Yoshiya, Koichi Noguchi

**Affiliations:** 1 Department of Anatomy and Neuroscience, Hyogo College of Medicine, 1-1 Mukogawa-cho, Nishinomiya, Hyogo 663-8501, Japan; 2 Department of Orthopaedic Surgery, Hyogo College of Medicine, 1-1 Mukogawa-cho, Nishinomiya, Hyogo 663-8501, Japan; 3 Department of Physiology, School of Dentistry, Nihon University, Kanda-Surugadai, Chiyoda-ku, Tokyo 101-8310, Japan; 4 Institute for Biomedical Sciences of Pain, Capital Medical University, Beijing 100069, P.R. China

## Abstract

The phosphorylation of extracellular signal-regulated kinase (pERK) in DRG and dorsal horn neurons is induced by the C-fiber electrical stimulation to the peripheral nerve. The present study was designed to investigate the expression and modulation of pERK in the rat dorsal horn neurons produced by repetitive electrical stimulation, and its involvement in the electrophysiological activity of dorsal horn neurons. Electrical stimulation of C-fiber intensity at different frequencies was applied to the sciatic nerve; the stimuli-induced pERK expression and the activity in dorsal horn neurons were studied by immunohistochemistry and extracellular recording, respectively. Electrical stimulation of C-fibers (3 mA) induced pERK expression in dorsal horn neurons in a frequency-dependent manner, indicating that the frequency of electrical stimulation is an important factor which activates the intracellular signal pathway in the spinal cord. To demonstrate the underlying mechanism of this frequency-dependent pERK expression, an NMDA receptor antagonist, MK-801, and a voltage sensitive calcium channel antagonist, nifedipine, were administrated intrathecally before the stimulation. We found that high frequency (0.5 Hz and 10 Hz) but not low frequent (0.05 Hz) stimulus-evoked pERK was partially inhibited by MK-801. Both high and low frequency stimulus-evoked pERK were inhibited by the nifedipine treatment. The extracellular single unit activities were recorded from the laminae I-II and V of the L4-5 dorsal horn, and we found that blockage of the intracellular ERK signal suppressed the wind-up responses in a dose-dependent manner. In contrast, any change in the mechanically evoked responses was not observed following the administration of ERK inhibitor. These observations indicate that ERK activation plays an important role in the induction of the wind-up responses in dorsal horn nociceptive neurons.

## Background

Extracellular signal-regulated kinase (ERK) is one of the mitogen-activated protein kinases (MAPKs) that transduce extracellular stimuli into intracellular post-translational and transcriptional responses [1,2]. They are activated by membrane depolarization and calcium influx [3], activated by an upstream kinase, MAPK/ERK kinase (MEK) [4], and known to be one of the intracellular signaling pathways involved in neuronal plasticity [2,5-7]. In nociceptive pathway, activation of high-threshold C-fibers by peripheral noxious stimuli causes not only an immediate sensation of pain, but also an increased responsiveness of neurons in the spinal dorsal horn that outlasts the initiating stimulus. Within a minute of an intense noxious peripheral or C-fiber electrical stimulus, many phosphorylated ERK (pERK) positive neurons were observed in lamina I and II of the dorsal horn [8]. A number of studies have suggested that the changes in MAPK cascades have an important role in neuroplastic changes in nociceptive pathways. In the mechanism of ERK activation in the dorsal horn, the synaptic transmission between primary afferents and dorsal horn neurons, especially regarding calcium influx, should be important, because calcium entry into neurons *via *ionotropic glutamate receptors may initiate the ERK signaling cascade [8,9].

We have demonstrated previously that activation of ERK in DRG neurons is dependent on the frequency of electrical stimulation at the C-fiber level [10]. In the present study, we found the frequency-dependent ERK activation also occurred in the spinal cord, and examined the pharmacological properties of this ERK activation. In an extracellular single-unit recording from dorsal horn neurons, the functional role of the ERK activation in dorsal horn was explored with the blockage of the intracellular ERK signals.

## Methods

Adult male Sprague Dawley rats (200–250 gm) were used. All animal experimental procedures were approved by the Committee on Animal Research at Hyogo College of Medicine and Nihon University School of Dentistry, and were performed in accordance with the National Institutes of Health guidelines on animal care. The animals were treated according to the guidelines of the International Association for the Study of Pain [11]. All efforts were made to minimize the number of animals used and their suffering.

### Electrical stimulation

All experimental procedures were done on rats that were deeply anesthetized with sodium pentobarbital (50 mg/kg, i.p.). Sterile operating instruments were used and special care was taken to prevent infection and to minimize the influence of inflammation. The left sciatic nerve was exposed and completely isolated from the surrounding connective tissue. Bipolar platinum wire electrodes were placed under the isolated sciatic nerve just distal to the level of the joint of the three terminal branches (the sural, common peroneal, and tibial nerves). Paraffin oil was pooled around the wire electrodes. After 20 min the sciatic nerve was hooked up, a train of 16 pulses of 3 mA, 2 ms, 0.05 Hz/0.5 Hz/10 Hz stimulation (Electronic Stimulator SKN-3201) to the sciatic nerve was delivered. Sham operations without electrical stimulation were also performed.

### Intrathecal administration of drugs

For the intrathecal administrations, a soft tube (CLAY ADAMS Brand, INTRAMEDIC polyethylene tubing, outer diameter, 0.61 mm) was implanted into the intrathecal space of the spinal cord (L4-L5 spinal cord segment) 2 days before the experiment. The end of tube was coated with bond and was implanted under the neck skin. The awake rats implanted with a tube ate, drank, and groomed normally and had no motor or sensory impairments. Drugs were injected 20 min before electrical stimulation. The NMDA receptor antagonist, MK-801 (1.5 nmol in 10 μl saline; n = 4), and the L-type calcium channel blocker, nifedipine (1.0 mM in 30% DMSO that was dissolved in artificial cerebrospinal fluid; 120 mM NaCl, 3 mM KCl, 1.2 mM MgCl_2_, 2.4 mM CaCl_2_, 23 mM NaHCO_3_, 1.2 mM NaH_2_PO_4_, 11 mM glucose pH 7.2~7.4; n = 4) was administered, as well as the vehicles.

### Immunohistochemistry

After appropriate survival times (the survival time after stimulation in all experiments was 2 min), rats were perfused transcardially with 1% paraformaldehyde in 0.1 M phosphate buffer followed by 4% paraformaldehyde in 0.1 M phosphate buffer, pH 7.4. The L4-L5 spinal cord was removed. Transverse spinal cord sections (18 μm) were cut and processed for pERK immunohistochemistry according to our previous studies [10]. The polyclonal primary antibody for pERK (New England BioLabs, Beverly, MA) at 1:1000 was used for DAB staining. For double immunofluorescence study, a combination of anti-pERK and the monoclonal primary antibody for NeuN (1:1000; Chemicon, Temecula, CA, USA) was used.

### Quantitative and statistical analysis for immunohistochemistry

For quantification of the immunoreactive profiles, eight sections from the L4-L5 lumbar spinal cord were selected randomly from each rat. The sections were then examined under light-field microscopy at X20 to localize immunopositive cells. The number of pERK positive neurons in the superficial laminae I-II and deep laminae III-IV was counted. As a stereological approach was not used in this study, thus quantification of the data may represent a biased estimate of the actual number of cells. Data were analyzed using a *t *test or analysis of variance (ANOVA) followed by Fisher's protected least-significant difference (PLSD) test. A difference was accepted as significant if *p *< 0.05.

### Animal preparation for single neuron recordings

Extracellular recording was performed on 12 Sprague Dawley rats (250–350 g). Rats were anesthetized with sodium pentobarbital (50 mg/kg, i.p.) and the trachea and right femoral veins were cannulated to allow artificial respiration and intravenous administration of drugs, respectively. L4-5 laminectomy was performed and bipolar silver wire electrodes were placed in the sciatic nerve. During recording sessions, anesthesia was maintained with halothane (2–3%) mixed with air (0.1 L/min) and rats were immobilized with pancuronium bromide (1 mg/kg/h, i.v.) and ventilated artificially. The expired CO_2 _concentration, rectal temperature and the electrocardiogram were monitored.

### Stimulation and single neuron recordings

The dorsal horn neurons were searched by applying mechanical stimulation (pressure or brush) to the skin of the hip and leg region. When a single neuron was isolated, the responses to mechanical stimulation were carefully examined and the receptive field was mapped. Then, graded punctate stimulation, brush and pinch stimuli were applied to the most sensitive areas of the receptive fields with von Frey filaments, camel brush and small arterial clip, respectively. After the identification of nociceptive neurons, electrical pulses with 2 ms and 2 mA were applied to the sciatic nerve. When the evoked responses with more than 90 ms latency could be recorded, repetitive stimulation (0.5 Hz) were tested whether the graded increase in firing was obtained. After that, 10 μl of MAPK kinase (MEK) 1/2 inhibitor U-0126 (Calbiochem, La Jolla, CA; 0.0025 M, 0.025 M and 0.25 M) was topically applied to the dorsal horn surface. The mechanical evoked responses were analyzed after drug administration. In order to avoid sensitization of the receptive field, we tested 1 or 2 nociceptive neurons with different receptive fields in each rat.

### Data analysis for single neuron recordings

Peristimulus time histograms (bin-width = 100 ms) were generated in response to each stimulus. Stimulus-response (S-R) functions of each nociceptive neuron were obtained in response to the mechanical (1, 6, 15, 26, 60 g), pinch and brush stimulation. Mean firing frequency for 10s during mechanical stimulation was measured. Electrical evoked responses were classified as A-fiber, C-fiber and afterdischarge according to the criteria of Seagrove et al. [12]. C-fiber evoked responses and afterdischarges were combined and were tested for an effect of U-0126 administration. In order to define the wind-up phenomena in nociceptive neurons following repeated electrical stimulation of the sciatic nerve, the evoked response following the first electrical stimuli (input response) was subtracted from the following 15 evoked responses. We tested U-0126 on only one nociceptive neuron recorded from one rat. Statistical analysis was performed using ANOVA followed by Fisher's PLSD test. Differences were considered significant at *p *< 0.05. Results are presented as means ± S.E.

## Results

**Figure 1:**
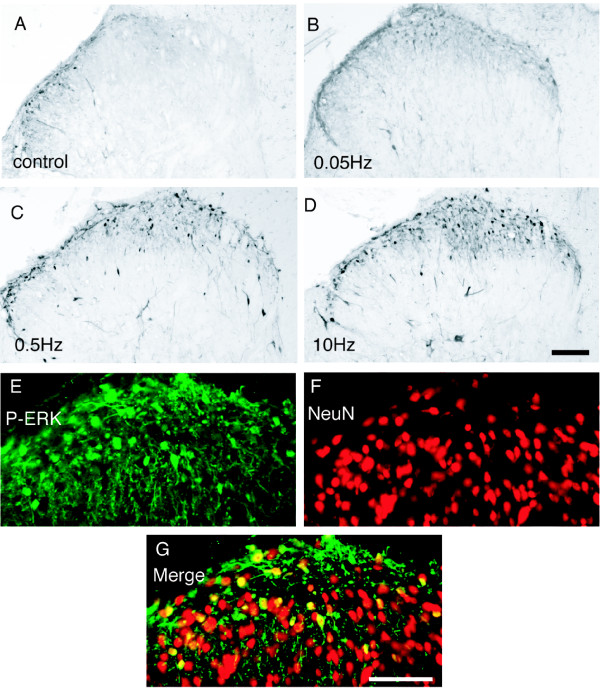
Expression of pERK in the ipsilateral L4/5 dorsal horn after electrical stimuli was frequency-dependent. A-D: Photomicrographs show pERK expression in spinal cord of rats that received sham operations without electrical stimulation (A), or 16 pulses of 3 mA electrical stimulation at frequencies of 0.05 Hz (B), 0.5 Hz (C), and 10 Hz (D) to the sciatic nerve. E-G: Double-immunofluorescence for pERK (*green, E*) and NeuN (*red, F*) after electrical stimulation at 3 mA/0.5 Hz to the sciatic nerve. Double staining appears yellow (G). Scale bars, 100 μm.

**Figure 2:**
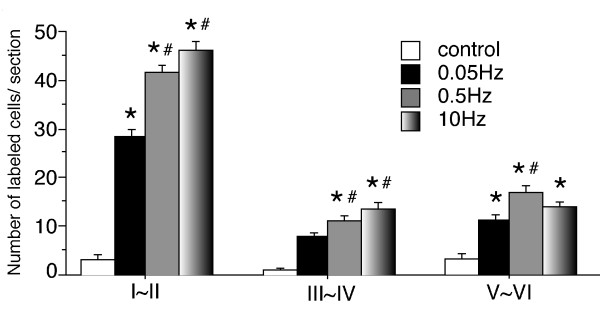
Number of pERK-ir profiles in lamina I-II, III-IV and V-VI of ipsilateral spinal cord after electrical stimulation at the indicated frequencies (n = 4 in each group, *p < 0.01 compared with control, ^#^*p *< 0.01, compared with 0.05 Hz).

Without any stimulation of the sciatic nerve, a small number of pERK immunoreactive (pERK-ir) profiles was observed only in the spinal lamina I-II posterior cutaneous nerve territory (Fig. 1A). The posterior cutaneous territory is thought to receive projections from the hip skin that was incised upon exposing the sciatic nerve. Thus, we focused on the pERK labeling only in the sciatic nerve territory of the dorsal horn. Electrical stimulation at 0.05 Hz to the sciatic nerve induced a small number of pERK-ir profiles, not only in superficial lamina, but also in deep laminae (Fig. 1B). The higher frequency electrical stimulation (0.5 Hz, 10 Hz,) induced a clear increase in pERK-ir profiles in the superficial laminae compared to the 0.05 Hz stimulus (Fig. 1C, D). To define cell type of the pERK-ir profiles, a double immunofluorescent staining was performed. The 0.5 Hz (3 mA, 2 ms) electrical stimulation-induced pERK expression co-localized with the NeuN positive cells (Fig. 1E–G), indicating ERK was activated mainly in the spinal neurons after the electrical stimulation. The quantification of labeled neurons in Fig. 2 indicated that the number of pERK-ir profiles induced by high frequency stimulation (0.5 Hz and 10 Hz) was significantly higher than that induced by the low frequency stimulation (0.05 Hz). Thus, the increase of pERK expression was dependent with the frequency of the C-fiber electrical stimulation. However, no significant difference was found between the pERK-ir profiles induced by 0.5 Hz and 10 Hz stimulation (Fig. 2).

**Figure 3:**
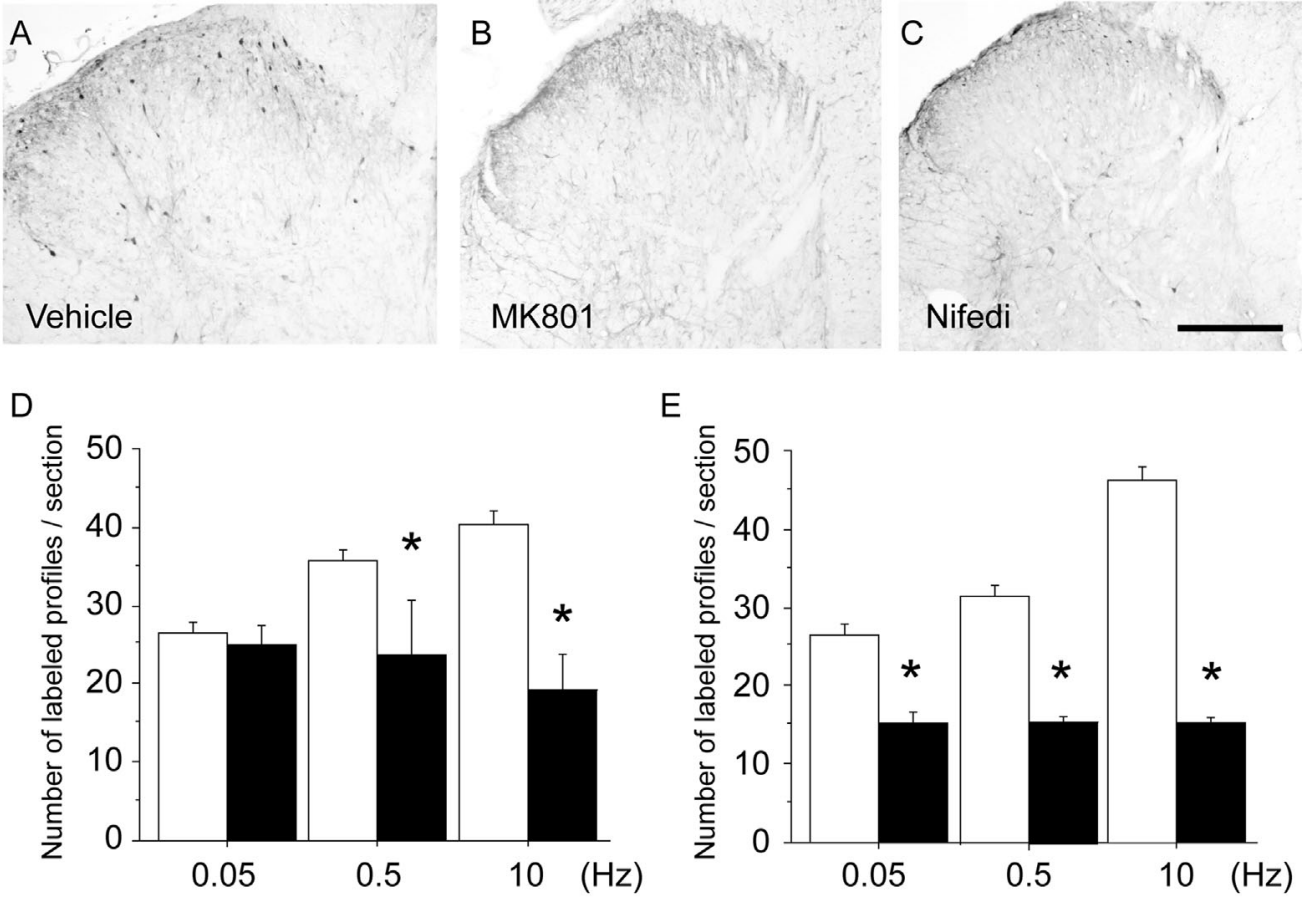
Intrathecal injection of MK-801 or nifedipine inhibited the frequency-dependent activation of pERK in the spinal dorsal horn. MK-801 or nifedipine was injected into the intrathecal space 20 min before the electrical stimulations. A-C: Photomicrographs show pERK expression evoked by electrical stimulation at 0.5 Hz in the spinal cord of rats that received vehicle (A), MK801 (B) and nifedipine (Nifedi, C). Scale bar, 100 μm (in C for A-C). D-E: Quantification of pERK immunoreactivity in lamina I-II of ipsilateral spinal cord after electrical stimulation at the indicated frequencies in the MK-801 treated group (D) and nifedipine treated group (E) (n = 4 in each group, *p < 0.01 compared with vehicle). Open bars; vehicle treated group, filled bars; MK-801 or nifedipine treated group.

Calcium entry into neurons *via *ionotropic glutamate receptors may initiate the ERK signaling cascade. We thus examined the involvement of the NMDA receptor on frequency-dependent ERK activation in laminae I-II. Pretreatment of intrathecal MK-801 injection did not inhibit the number of pERK-ir profiles after low frequency (0.05 Hz) stimulation (Fig. 3D), while it clearly inhibited the spinal ERK activation induced by high frequency (0.5–10 Hz) stimulation (Fig. 3A, B). The number of pERK-ir profiles induced by high frequency stimulation (0.5 Hz, 10 Hz) was significantly, but not completely, reduced in the spinal lamina I-II (Fig. 3D). These data suggested that ERK activation by high frequency electrical stimulation is at least partly involved in the NMDA receptor mechanism, whereas low frequency may not involve NMDA activation.

In consideration of synaptic transmission between primary afferents and dorsal horn neurons, we next examined the effect of the voltage sensitive calcium channel (VSCC) blocker, nifedipine, on the frequency-dependent pERK activation. Nifedipine is known as an L-type calcium channels blocker, and this type of calcium channel is thought to be postsynaptic and therefore depolarization of neurons would be sufficient to open this type of voltage-gated calcium channel. The pretreatment with nifedipine by intrathecal administration reduced the ERK activation in the spinal lamina I-II both in the low frequency (0.05 Hz) and high frequency (0.5 Hz and 10 Hz) stimulation (Fig. 3C). Statistically significant inhibitions were observed in the number of pERK-ir profiles (Fig. 3C, E).

**Figure 4:**
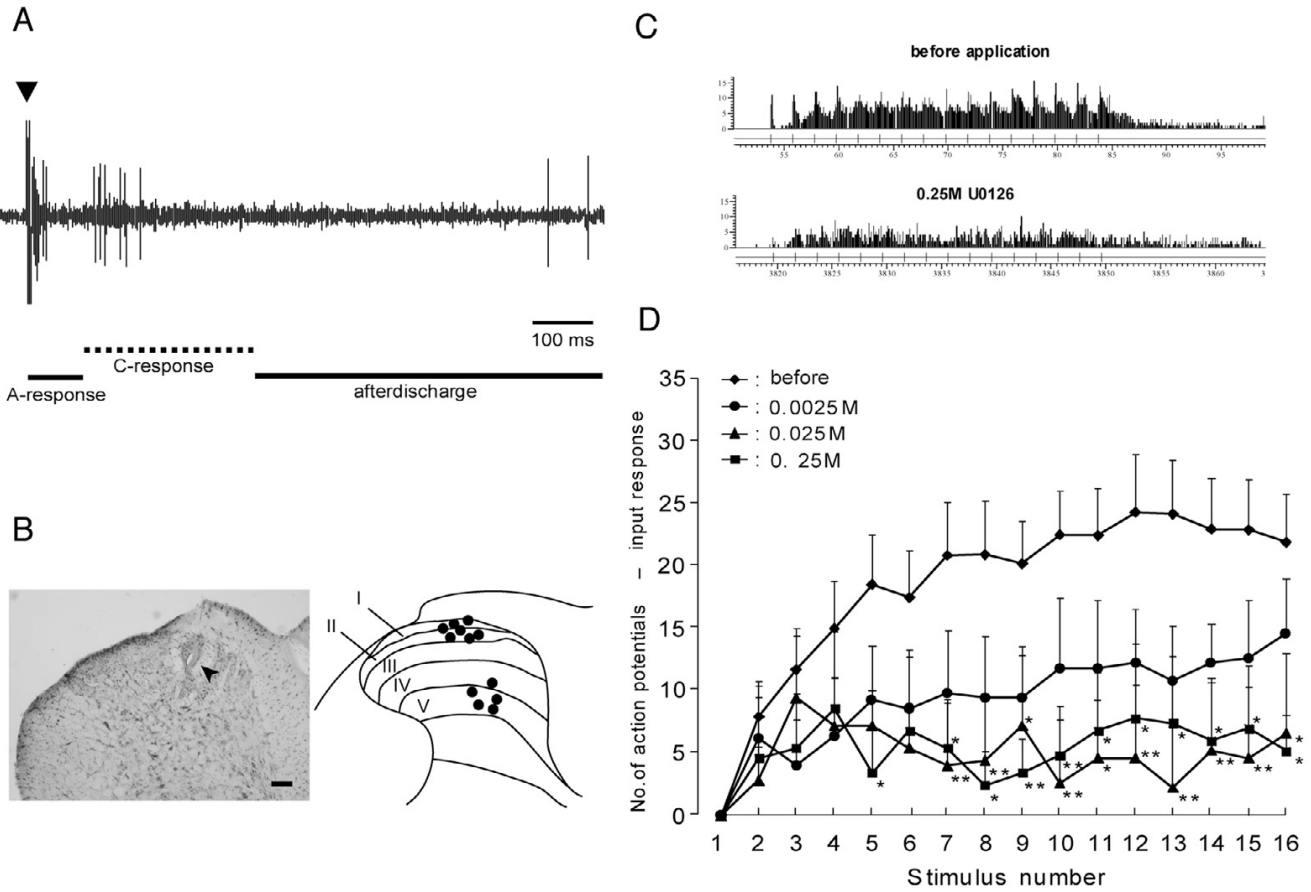
U-0126 administration depressed wind-up in dorsal horn nociceptive neurons. A: The typical response of the dorsal horn nociceptive neuron following electrical stimulation of the sciatic nerve (2 ms, 2 mA). B: A photomicrograph of the dorsal horn and the camera-lucida drawings of recording sites in the dorsal horn. An arrow head indicates the lesion by DC current application through the recording electrode. C: Typical responses of a dorsal horn nociceptive neuron following repetitive electrical stimulation of the sciatic nerve before (upper trace) and after (lower trace) U-0126 application. D: The effect of U-0126 administration on the mean evoked responses of dorsal horn nociceptive neurons following repetitive electrical stimulation of the sciatic nerve (n = 12). Note that evoked responses were depressed following U-0126 administration in a dose-dependent manner (*: *p *< 0.05, **: *p *< 0.01).

In order to examine the functional role of ERK activation in the dorsal horn after electrical stimulation of the primary afferent, we recorded single neuronal activities from the dorsal horn. We have searched and identified nociceptive neurons that showed C-responses to electrical stimulation of the sciatic nerve (Fig. 4A). Those neurons were located in superficial and deep dorsal horn neurons (n = 7 and n = 5, respectively), shown in Fig. 4B. We found wind-up phenomenon in superficial and deep dorsal horn neurons following repeated electrical stimulation of the sciatic nerve (Fig. 4C). We compared the wind-up phenomenon before and after application of the ERK inhibitor, and found that the increased responses following repeated stimuli were significantly depressed after topical application of U-0126 (Fig. 4C). Only a slight depression of the evoked responses was observed at the 2^nd ^to 4^th ^trials, whereas a significant depressive effect of increased responses was observed at following the 5^th ^to 16^th ^trials as illustrated in Fig. 4D. The depressive effect by U-0126 was dose dependent (Fig. 4D). These data suggest that pERK may have a role on cumulative depolarization after repetitive C-fiber stimulation, thus causing wind-up.

**Figure 5:**
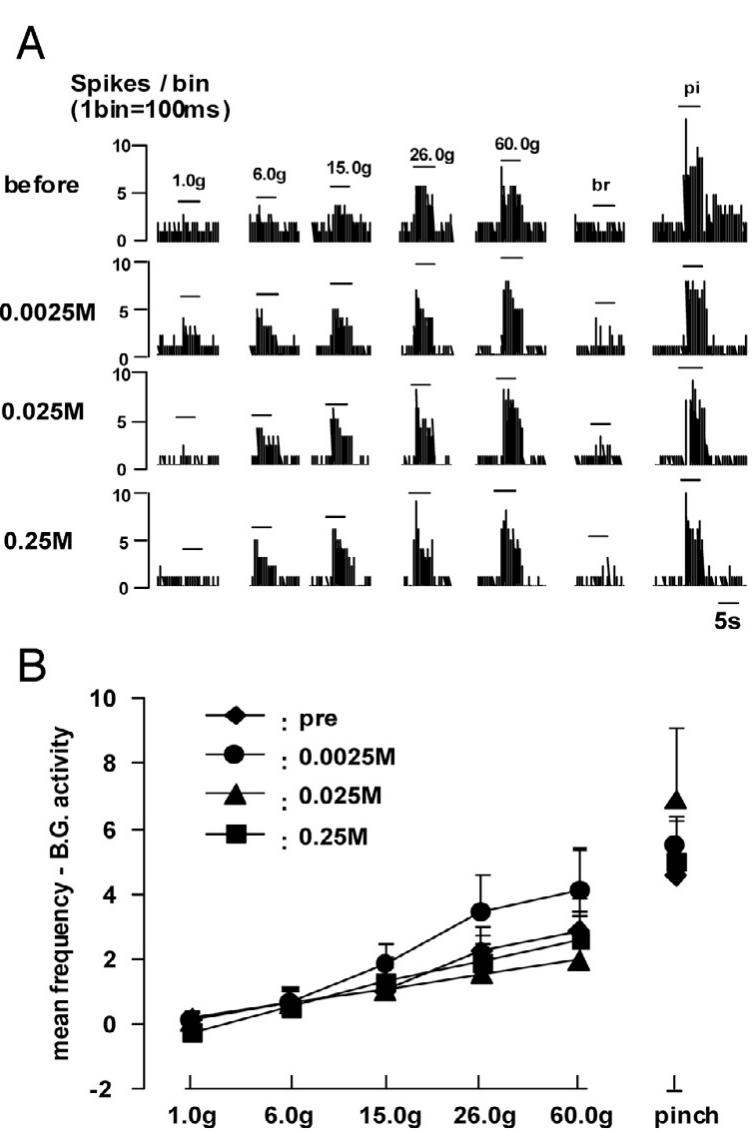
U-0126 administration does not affect mechanical evoked responses in DH nociceptive neurons. A: Typical responses of a DH nociceptive neuron following graded mechanical, brush and pinch stimuli. br: brushing of the receptive field, pi: pinching of the receptive field. B: Stimulus response functions of mechanical evoked responses in DH nociceptive neurons. Note that the evoked responses were not affected by U-0126 administration.

Next, we examined whether the activation of ERK in dorsal horn neurons may have a role on the evoked responses by the natural stimuli to the receptive field. Typical examples of the evoked responses of WDR neuron following graded mechanical, brush and pinch stimuli are illustrated in Fig. 5A. Each neuron gradually increased its firing frequency following increases in mechanical stimulus intensity. The experiment in which U-0126 was administered intrathecally revealed that the mechanically evoked responses were not affected by U-0126 administration (Fig. 5A). Quantitative data showed no statistically significant difference among any dose of U-0126 and pre-value (Fig. 5B).

## Discussion

In the present study, we described the frequency-dependent phosphorylation of ERK in dorsal horn neurons by electrical stimulation at C-fiber intensity and its involvement in electrophysiological activity of dorsal horn neurons. Antagonists of NMDA receptors and VSCCs, which depress the wind-up response [13-17], inhibited the ERK phosphorylation. Furthermore, U-0126, an ERK signal inhibitor, dose-dependently depressed the wind-up response evoked by the repetitive electrical stimulation of the peripheral nerve. However, any change in the mechanically evoked responses was observed following U-0126 administration.

In order to understand the mechanism of frequency-dependent ERK activation in the dorsal horn by electrical stimulation, we focused on the synaptic transmission between primary afferents and dorsal horn neurons, especially regarding calcium influx. Calcium entry into neurons *via *ionotropic glutamate receptors is known to activate ERK signaling cascade [8,9]. Our experiment showed that the effect of the NMDA receptor antagonist on ERK activation is frequency-dependent. The features of the frequency-dependent ERK activation in dorsal horn neurons, at least in part, are similar to the electrophsiological properties, such as wind-up phenomenon in the dorsal horn neurons. Wind-up is a frequency-dependent phenomenon that is triggered at a critical frequency of activation of afferent C-fibers [18]. Below the critical frequency of 0.2–0.3 Hz, wind-up is not observed, and above frequencies of 20 Hz habituation of the response or wind-down is typically observed [19]. A number of studies have indicated that enhanced wind-up may reflect changes in the central synapses of C-fibers and the postsynaptic neurons that reflect temporal summation of slow cumulative depolarizations in the dorsal horn. The NMDA receptor is a target receptor involved in the wind-up phenomena in dorsal horn nociceptive neurons [15,20].

The ERK signaling cascade is a key pathway that mediates NMDA receptor-dependent neuronal plasticity [21]. One potential mechanism of this regulation by ERK is indirect, by long-term modulation of cell properties through the control of gene transcription and regulation of channel gene expression, which is unlikely the mechanism for the short-term wind-up. Another possible mechanism by which ERK might modulate neuronal excitability is through direct regulation of membrane ion channels that regulate the membrane potential and thereby intrinsic membrane properties. Proposed channels targeted by ERK include the K channel [22,23], the Na channel [24] and the Ca channel [25,26]. Changes in these channels' activity may cause a cumulative depolarization after repetitive C-fiber stimulation, thus causing wind-up. Our data clearly showed that a MEK inhibitor, U-0126, inhibited the wind up dose-dependently, and the dose of U-0126 indeed inhibited ERK activation in spinal cord neurons, which was demonstrated in our previous studies [27,28] and many other reports [29-33].

Important point in the present study is the time course of the activation of ERK after stimulation. In almost all studies, including the present study, that examine pERK in the dorsal horn *in vivo*, the activation of ERK was detected first at 2 min after various external stimulus presentations [8,33]. This is entirely due to the minimum time necessary to perform the immunohistochemical procedures. The phosphorylation of ERK in the dorsal horn likely occurs more rapidly, i.e. in seconds or less than a minute. In fact, in our wind-up experiments, the effect of the MEK inhibitor was observed after several trials, 10–20 seconds after the start of train stimuli. Therefore, the phosphorylation of ERK may generate calcium entry to the spinal cord very rapidly, within seconds, which could synchronize with the wind-up. Another point we have to consider is that a majority of ERK positive neurons were concentrated in laminae I-II of the dorsal horn, however single neuronal activities showed wind-up responses in superficial and deep dorsal horn neurons. It is difficult to know the exact causal link between ERK activation and the wind-up phenomenon, especially about the discrepancy of location and the very early time points after external stimulation *in vivo*. We also noticed that the inhibition produced by NMDA receptor antagonist was not complete. The data suggest other mechanisms in addition to calcium entry through the NMDA receptor ion channel may be responsible for initiating an activation of the ERK cascade, for example VSCCs. Although stimulation in any frequency can induce the phosphorylation of ERK, only high frequency electrical stimulation (0.5 Hz and 10 Hz) activates NMDA receptors that seem capable of promoting VSCCs activation in inducing the pERK stain. These features also can be seen in the wind-up, for example, blockade of wind-up by NMDA receptor antagonists is only partial [13,15]; the nifedipine treatment also depresses wind-up [16,17].

We described the frequency-dependent phosphorylation of ERK in dorsal horn neurons and its involvement in wind-up phenomena in dorsal horn neurons. In contrast, ERK activation did not have any role in the mechanically evoked responses following U-0126 administration. It is possible that the long lasting high frequency-strong stimuli generate barrage of action potentials in the dorsal horn neurons, resulting in the abnormal activation of the dorsal horn nociceptive neurons. The mechanism may be similar to that was reported in inflammation or nerve injury-induced abnormal activation of dorsal horn neurons. It is highly likely that ERK activation contributes to produce hyperexcitability of dorsal horn nociceptive neurons under these excitable conditions.

## Abbreviations

ANOVA, analysis of variance; ERK, extracellular signal-regulated kinase; LTP, long-term potentiation; MAPK, mitogen-activated protein kinase; NS, nociceptive specific; PLSD, protected least-significant difference; S-R, stimulus-response; VSCC, voltage sensitive calcium channel; WDR, wide-dynamic-range.

## Competing interests

The author(s) declare that they have no competing interests.

## Authors' contributions

TF, YD: Major data collection; data analysis, paper writing

SW, XC: Morphological data collection

HY, KO: KK, Data interpretation

KI, HK: Electrophsiological data collection, paper writing

SY, KN: Project conception and design, paper writing
